# Influence of co-cultures of *Streptococcus thermophilu*s and probiotic lactobacilli on quality and antioxidant capacity parameters of lactose-free fermented dairy beverages containing *Syzygium cumini* (L.) Skeels pulp†

**DOI:** 10.1039/c9ra08311a

**Published:** 2020-03-10

**Authors:** Sabrina Laís Alves Garcia, Gabriel Monteiro da Silva, Juliana Maria Svendsen Medeiros, Anna Paula Rocha de Queiroga, Blenda Brito de Queiroz, Daniely Rayane Bezerra de Farias, Joyceana Oliveira Correia, Eliane Rolim Florentino, Flávia Carolina Alonso Buriti

**Affiliations:** Post-Graduate Program on Pharmaceutical Sciences, Centre of Biological and Health Sciences, State University of Paraíba R. Juvêncio Arruda, s/n 58429-600 Campina Grande PB Brazil flavia@ccbs.uepb.edu.br flavia.carolina@pq.cnpq.br; Centre of Research and Extension on Food, Centre of Sciences and Technology, State University of Paraíba R. Juvêncio Arruda, s/n 58109-790 Campina Grande PB Brazil; Department of Chemistry, Centre of Sciences and Technology, State University of Paraíba R. Juvêncio Arruda, s/n 58109-790 Campina Grande PB Brazil; Department of Pharmacy, Centre of Biological and Health Sciences, State University of Paraíba R. Juvêncio Arruda, s/n 58429-600 Campina Grande PB Brazil

## Abstract

This study investigated the influence of probiotic lactobacilli in co-culture with *Streptococcus thermophilus* on composition, physicochemical parameters, microbial viability, sensory acceptability, antioxidant capacity and protein profile of lactose-free fermented dairy beverages with added whey, β-galactosidase and jambolan (*Syzygium cumini* (L.) Skeels) pulp. Three beverages (T1, T2 and T3) were prepared with *Streptococcus thermophilus* TA-40 as starter culture. *Lactobacillus rhamnosus* LR32 and *Lactobacillus casei* BGP93 probiotic cultures were added into T2 and T3, respectively. The probiotic adjuvants slightly influenced the pH and titratable acidity of dairy beverages, with no influence on the proximate composition and on the sensory attributes. Samples presented fat and protein contents suitable to meet the requirements of “low-fat dairy beverages with non-dairy ingredients added” according to the Brazilian legislation, lactobacilli viability above 7 log CFU g^−1^ for both probiotics and total phenolic content around 40 mg GAE 100 g^−1^. Colour was the most evaluated sensory aspect (average scores close or higher than 8 in a scale from 0 to 10 for most of the sampling periods). The overall antioxidant capacity increased significantly following the addition of jambolan (*p* < 0.05), and significantly more during storage (*p* < 0.05), likely due to proteolysis verified in the electrophoresis gels, as a result of the metabolism of the lactic cultures. The dairy beverages studied are good options for functional foods due to their nutritional value, viability of probiotic lactobacilli, phenolic content, and antioxidant capacity, also serving lactose-intolerant people.

## Abstract 2

Probiotic dairy beverages for lactose intolerants had lactobacilli viability above 7 log CFU for 21 days. The co-cultures studied showed proteolytic activity and reinforced the effect of the jambolan pulp on the antioxidant capacity of the products.

## Graphical abstract

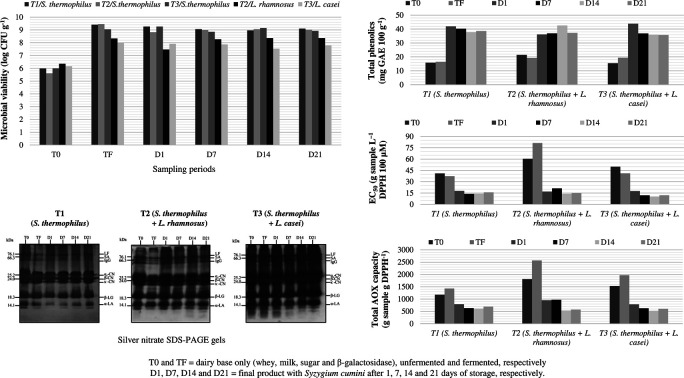

## 1. Introduction

The food habits and lifestyles of the population are increasingly following the ideas of the food market, which strengthens the responsibility of the industry to bring more attractive foods from sensorial and nutritional points of view.^1^ Among these foods, the so-called functional foods stand out, which are able to exert beneficial effects on one or more target organ, thus contributing to the health and well-being of the consumer.^2^

On the other hand, it is believed that a large number of diseases may be directly related to reactive oxygen species, more commonly called free radicals, which are produced during aerobic metabolism.^3^

Based on the study conducted by Alenisan and co-workers,^4^ it is understood that dairy products in general are potentially promising foods with respect to antioxidant activity, since milk contains antioxidant molecules traces, which are transferred from the animal diet, and also casein and whey proteins, which can release free radical inhibitors after proteolysis. Moreover, according to the authors, the nutritional and therapeutic properties of dairy products can be improved by the addition of plant sources rich in phenolic compounds.^4^ Phenolic compounds play a protective role against the action of free radicals, suggesting a reduced risk of chronic diseases and increased health benefits.^3^ In general, free radical inhibitors can also prevent the peroxidation of food products, increasing their shelf life and antioxidant capacity.^4^

As pointed out by dos Santos and co-workers,^5^ dietary polyphenols also have a close relationship with the intestinal microbiota, since these compounds require the metabolic action of those microorganisms to become active, while polyphenols select the intestinal microbiota directly, influencing the modulation of the gastrointestinal microbial population. The authors also emphasize that the composition of intestinal microbiota can be improved by the consumption of probiotic microorganisms and that dairy products have been the primary option of probiotic carriers.^5^

Jambolan (*Syzygium cumini* (L.) Skeels), a fruit still little explored in Brazil and with no stablished market, have been reported as a good source of polyphenols such as phenolic acids, tannins and flavonoids, particularly anthocyanins, being an interesting option to be used in the development of probiotic dairy products.^6,7^

However, it is estimated that more than 70% of the world population suffers from some degree of lactose intolerance.^8,9^ Lactose-free dairy products can be obtained by the treatment of milk with the commercial enzyme lactase (β-galactosidase), which is produced by microorganisms of the genera *Aspergillus* and *Kluyveromyces*.^9^ The hydrolysis of lactose and the use of probiotic bacteria are alternatives for obtaining a functional food that does not present restrictions in consumption to individuals who are intolerant to this disaccharide.

Furthermore, the costs of fermented and unfermented dairy foods are reduced with the use of whey in their production chains since it is a by-product of the manufacture of cheese.^10,11^ Whey protein-based ingredients has been described to be protective towards probiotic bacteria in food products, increasing the viability of these microorganisms throughout the product shelf life.^12^ Moreover, probiotic cultures added to food products formulated with whey, particularly the dairy beverages, may favour the release of bioactive peptides from whey proteins.^13^

Considering the opportunities of multiple benefits offered by a lactose-free probiotic dairy product with the use of whey and a tropical fruit source of phenolic compounds, this study aimed to investigate the influence of probiotic cultures of *Lactobacillus casei* and *Lactobacillus rhamnosus* on composition, physicochemical parameters, microbial viability, sensory acceptability, antioxidant capacity and protein profile of dairy beverages added with whey, β-galactosidase and jambolan pulp.

## 2. Materials and methods

### 2.1. Obtaining the jambolan fruits

The jambolan fruits were collected in the city of Lagoa Seca (07° 10′ 15′′ S, 35° 51′ 14′′ W, 634 m height), state of Paraíba, Brazil, during their harvest period in February 2017. The fruits were washed in running water and sanitised with sodium hypochlorite solution at a concentration of 200 mg L^−1^ free chlorine. Manual pulping, grinding, and pasteurisation of the pulps were performed, followed by immediate freezing at −18 °C.

### 2.2. Processing of cheese to obtain whey

The whey was obtained from the processing of Minas Frescal cheese, as described by Almeida Neta and co-workers.^10^ Pasteurised skimmed milk (Cariri Light, Cooperativa Agropecuária do Cariri, Campina Grande, Brazil) was added to calcium chloride (Neon Comercial, São Paulo, Brazil, 0.25 g L^−1^ milk) and the coagulant Hannilase® (Chr. Hansen, Valinhos, Brazil), according to manufacturer's instructions. The milk was heated to 34–37 °C for the addition of other ingredients. The milk was homogenised and rested for approximately 45 min until complete coagulation, after which the curd was cut. Molding of the cheese was performed, and the whey was drained into plastic bottles and stored at −18 °C until use.

### 2.3. Preparation of lactose-free dairy base

Sucrose (Estrela, Biosev, Arês, Brazil, 80 g) was solubilised in 840 mL whey (previously obtained from Minas Frescal cheese) and heat-treated at 85 °C for 5 min. Thereafter, 80 g skimmed milk powder (Molico, Nestlé, Araçatuba, Brazil) was added, subjected to heat treatment for 30 min at 85 °C, and immediately cooled to room temperature. For the hydrolysis of lactose, the enzyme β-galactosidase (Prozyn® Lactase, Prozyn, Brazil, 0.50 g L^−1^ dairy base) was added and refrigerated at 4 °C for 24 h. To evaluate the effectiveness of lactose hydrolysis, the content of this disaccharide in the dairy bases following β-galactosidase treatment was determined through high-performance liquid chromatography (HPLC) using a refractive index detector at Embrapa Agroindústria de Alimentos (Rio de Janeiro, RJ, Brazil).^14^ The result was lower than the limit of detection of the method (<100 mg/100 g, data not shown), being in agreement with the products classified as lactose-free according to the current Brazilian legislation.^15^

### 2.4. Production of the lactose-free fermented dairy beverage

The dairy base was heated to 43 ± 1 °C for the addition of the cultures. The starter culture of *Streptococcus thermophilus* TA 40 (TA 40 Lyo 50 DCU, Yo-mix™, Danisco France SAS, Sassenage, France) was added at a concentration of 0.003 g/100 g in all trials (TI, T2, and T3), and the commercial probiotic cultures of *Lactobacillus rhamnosus* LR32 (LR32 200 B 100 GM, FloraFit® Probiotics, Danisco USA Inc., Madison, WI, USA) and *Lactobacillus casei* BGP93 (Lyofast BGP93, 5 Doses, Sacco SRL, Cadorago, Italy) were added at a concentration of 0.02 g/100 g in trials T2 and T3, respectively. Information on the main characteristics of the probiotic strains *L. rhamnosus* LR32 and *L. casei* BGP93 was summarized in another study.^16^ The dairy bases were fermented at 43 ± 2 °C until an acidity greater than 0.7 g/100 g lactic acid was obtained. Following fermentation, the pulp of *S. cumini* was added at 15 g/100 g.

### 2.5. Determination of the composition of the fermented dairy beverage

The total solids, ash, fat, and protein contents of the dairy beverage were determined in triplicate for three batches of each beverage on the first day of storage. Total solids were determined by drying 2 g sample at 70 °C in a vacuum oven (Marconi, model MA 030/12, Piracicaba, Brazil).^17^ The ash content was determined gravimetrically by incinerating the dried samples at 550 °C.^17^ The fat content was determined according to the method described by Folch and co-workers.^18^ The protein content was estimated by measuring the nitrogen contained in 0.2 g sample using the micro-Kjeldahl method and multiplying by a conversion factor of 6.38.^19^ The total carbohydrate content was obtained by the difference in reaching 100 g/100 g of the total composition.^20^

### 2.6. Physicochemical parameters and viability of the starter culture and adjuvants

The pH, titratable acidity, and viability of the starter culture (*S. thermophilus*) and probiotics (*L. rhamnosus* and *L. casei*) were evaluated in triplicate before and after fermentation (T0 and TF), on the first day (D1), and after 7, 14, and 21 days of storage (D7, D14, D21, respectively). The pH of the samples was evaluated using a Tecnal pH meter (model TEC 3P MP, Piracicaba, Brazil). The titratable acidity was determined according to the official method and expressed in terms of g/100 g^−1^ lactic acid.^17^ For the microbiological analysis, a 25 g sample was weighed, aseptically transferred to 225 mL sterile NaCl solution (8.5 g L^−1^), and subjected to serial dilutions with the same diluent. *S. thermophilus* populations were determined by the addition of 1 mL each dilution to M17 agar (Difco, Sparks, MD, USA) containing lactose (Vetec, Duque de Caxias, Brazil, 5 g L^−1^), followed by incubation at 37 °C for 48 h.^11^ Populations of *L. rhamnosus* and *L. casei* were determined by the addition of 1 mL each dilution to acidified MRS (Acumedia, Lansing, MI, USA) agar (adjusted to pH 5.4 with acetic acid), followed by incubation at 37 °C for 72 h.^11^

### 2.7. Sensory evaluation of the fermented dairy beverage

The sensory evaluation used in the present study was approved by the Ethics Committee of the State University of Paraíba, Paraíba (UEPB), Brazil, Certificate of Presentation for Ethical Assessment (CAAE) no. 2.229.0000.5187, and was performed in the Laboratory of Sensory Analysis at the Federal University of Campina Grande (UFCG), Paraíba State, Brazil. The dairy beverages were evaluated after 7, 14, and 21 days of storage through acceptability tests using the 11-point hybrid hedonic scale (0 = I did not like it at all, 5 = I neither liked nor disliked it, 10 = I liked it a lot),^21,22^ focussing on the attributes of flavour, consistency, appearance, colour, and general acceptability. One hundred and twenty-eight untrained judges; mainly students, professors, staff, researchers, and fellows from UFCG and UEPB, or other healthy volunteers, participated in the present study. The samples were kept under refrigeration before testing and were served monadically in plastic cups coded with three random digits. During one session, each consumer analysed three samples, which were served in a randomized order. The judges were also instructed to report the sensory attributes related to taste, sweetness, consistency, appearance, and colour that they liked and disliked about the samples, and they were free to mention no or more than one attribute. To ensure the safety of the judges, contaminant analyses of total coliforms (at 35 °C), thermotolerant coliforms (at 45 °C), and *Salmonella* sp. were carried out the day after the manufacture of the products intended for sensory analysis. The contaminant results showed that the dairy beverages were safe for consumers, since they had a count lower than 100 CFU g^−1^ for the analysis of coliforms at 35 °C (and therefore for coliforms at 45 °C) and the absence of *Salmonella* sp. in 25 g.^23^

### 2.8. Extraction of phenolics for the analysis of phenolic content and antioxidant capacity

The extracts of the bases and fermented dairy beverages were obtained according to the methodology proposed by dos Santos and co-workers,^5^ with some modifications. The milk bases were evaluated before and after fermentation and the dairy beverages after 1, 7, 14, and 21 days of storage. The samples were weighed using an analytical balance (±1.2500 g) and mixed with 5 mL acidified methanol (100 μL P.A. hydrochloric acid in 100 mL methanol). The samples were vortexed and stored for at least 12 h under refrigeration at 4 °C. The following day, the samples were centrifuged (centrifuge 5810R, Eppendorf, Hamburg, Germany) at 13 500 × *g* for 5 min at 4 °C. The residue was washed with methanol–HCl and centrifuged again. This procedure was repeated once more. The supernatants were used for the analyses.

### 2.9. Analysis of total phenolic content

The phenolic content of the fermented dairy beverage formulations containing jambolan pulp (T1, T2, and T3) was evaluated during the storage period (D1, D7, D14, and D21) and compared with that of the dairy bases before (T0) and after (TF) fermentation without the addition of jambolan. The analysis was performed with two batches of the product.

The total phenolic content was determined according to dos Santos and co-workers,^5^ with some modifications. All procedures were performed in the dark. Volumes of 60 μL each prepared extract, 2340 μL distilled water, and 150 μL Folin–Ciocalteau reagent (Sigma-Aldrich Chemie GmbH, Steinheim, Germany) were sequentially added to 15 mL plastic test tubes. Following an incubation period of 8 min, 450 μL Na_2_CO_3_ solution (Neon Commercial, Sao Paulo, Brazil, 30 g/100 mL) was added, mixed, and allowed to stand for 30 min at room temperature. The absorbance was measured at 750 nm using an SP-2000 UV spectrophotometer (Spectrum, Shanghai, China) as compared with a standard curve previously constructed using gallic acid (Vetec, Sigma-Aldrich, Duque de Caxias, Brazil). The results are expressed as mg gallic acid equivalent (mg GAE) per 100 g sample.

### 2.10. DPPH assay and calculation of the antioxidant capacity

The antioxidant capacity of the dairy beverages was assessed by the 1,1-diphenyl-2-picryl-hydrazyl (DPPH) radical scavenging assay in two batches using an adapted version of the protocol described by Rufino and co-workers.^24^ A volume of 50 mL DPPH stock solution was prepared by dilution of 0.0020 g DPPH (Adrich Chemistry, Sigma-Aldrich Co., St. Louis, MO, USA) in ethanol. Different volumes of the sample extracts (50 μL, 100 μL, and 200 μL) were mixed with 100 μM DPPH aliquots (2.95 mL, 2.90 mL, and 2.80 mL, respectively) to give a total volume of 3 mL. The control samples were prepared with ethanol instead of sample extract. The decrease in absorbance at 517 nm was measured after an incubation period of 60 min at room temperature. The results are expressed as percentage DPPH scavenging effect (%) using the following eqn (1):1
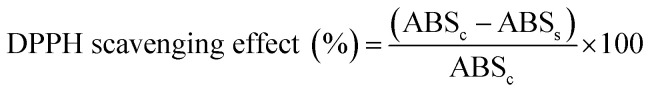
where ABS_c_ is the absorbance of the control with an aliquot of 200 μL of ethanol after 60 min (DPPH solution without the sample extract) and ABS_s_ is the absorbance of the sample with 200 μL extract after 60 min.

An inhibition concentration curve, constructed with the absorbances after 60 min of the samples with 50 μL, 100 μL, and 200 μL of extracts in DPPH solution, and a DPPH standard curve were used to calculate the amount of dairy beverage required to reduce the concentration of DPPH by 50% (EC_50_) in g sample per L of 100 μM DPPH initial solution.

The total antioxidant capacity, expressed as g DPPH per g sample, was obtained according to the following eqn (2), based on Rufino and co-workers:^24^2
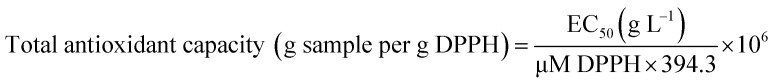
where EC_50_ (g L^−1^) is the amount of dairy beverage (g) required to reduce the initial concentration of DPPH per L by 50%, μM DPPH is the concentration of DPPH scavenged by the beverage extract that decreased the absorbance by 50% and 394.3 is the molar mass of DPPH.

### 2.11. Analysis of the protein profile of the fermented dairy beverages using the sodium dodecyl sulphate polyacrylamide electrophoresis (SDS-PAGE) technique

Gels were run for each treatment (T1, T2, T3), with each gel containing samples of the dairy bases before and after fermentation and the dairy beverage on days 1, 7, 14, and 21 of storage. Samples were denatured in 1 mL buffer prepared with 5 mL glycerol, 5 mL 10% sodium dodecyl sulphate (SDS, Sigma-Aldrich, St. Louis, MO, USA), 2.5 mL Tris–HCl (0.6 mol L^−1^, pH 6.8), 0.3 mL β-mercaptoethanol (Sigma-Aldrich), 2.5 mg bromophenol blue, and 25 mL (qsp) deionised water. Next, samples were shaken at 100 °C for 10 min (Tecnal, TE-393/2, Piracicaba, Brazil), and centrifuged (Parsec Biotechnik CT 0603 centrifuge, Curitiba, Brazil) at 3000 rpm for 2 min. The supernatants were discarded. The PAGE-SDS-2-mercaptoethanol system, described by Laemmli^25^ and Pereira and co-workers,^13^ adapted for the use of plate gels (10.5 × 10 × 0.4 cm) (mini vertical, model SE250, Hoefer, Holliston, MA, USA) was used. A 15% polyacrylamide separating gel and 5% stacking gel were used. The power supply was set at 200 V, and the gels were run at 60 mA per gel in a buffer solution consisting of 6.32% (w/v) glycine (Biorad, Hercules, CA, USA), 4.0% (w/v) Tris (Biorad), and 1% (w/v) SDS at pH 8.9. The gels were stained overnight in a solution of 0.01% (w/v) Coomassie brilliant blue (R-250; Biorad), 40% (v/v) methanol, and 10% (v/v) acetic acid, following by destaining in the same solution lacking the stain. The gels were then photographed using a scanner and stained with a solution of 0.2% (w/v) silver nitrate containing 74 μL formaldehyde per 100 mL. Subsequently, gels were sensitised in a solution of 0.02% sodium thiosulphate for 1 min and restained with silver nitrate solution. The gels were developed in 6% (w/v) sodium carbonate solution containing 50 μL formaldehyde and 2 mL 20% (w/v) sodium thiosulphate solution per 100 mL. Following development, the gels were photographed again using a scanner. The molar masses of protein bands lactoferrin (LF), serum albumin (SA), α_s_-casein (α_s_-CN), β-casein (β-CN), β-lactoglobulin (β-LG) and α-lactalbumin (α-LA) were assessed by comparing their relative mobilities with those reported by the sixth revision of nomenclature of the cow milk proteins.^26^

### 2.12. Statistical analysis

Data are presented as the mean ± standard deviation. Initially, the data were analysed for normality using the Shapiro–Wilk test, and homogeneity of variances using the Bartlett test. Following confirmation of these assumptions, the data were submitted to analysis of variance, and the means were compared using the Tukey test, with a significance level of 5%. The other data were analysed by means of nonparametric equivalence tests. Statistical analyses were performed using the Statistica 6.0 program (Statsoft Inc., Tulsa, OK, USA).

## 3. Results and discussion

### 3.1. Composition of the dairy beverage

The results of the mean composition on the first day of storage under refrigeration are shown in Table 1. There was no significant difference in the mean composition between the three beverages (*p* > 0.05), since the formulations differed only in the probiotic cultures, which did not interfere with the studied parameters. The beverages had protein content in accordance with the requisite established by the Brazilian regulatory standards for this type of product,^27^ since the content was higher than 1 g protein per 100 g fermented dairy beverage following the addition of food ingredients other than those of dairy origin. The average fat content was less than 0.5 g/100 g for all beverages, which may allow their classification as low-fat products, since the limit established in the Brazilian legislation is 3.0 g total fat per serving,^28^ or 200 g for dairy beverages.^29^

**Table 1:** Mean composition of fermented dairy beverage trials containing jambolan pulp on the first day of refrigerated storage at 4 ± 1 °C^a^

Parameters	Trials		
	T1	T2	T3
Total solids (g/100 g)	20 ± 1^A^	19.4 ± 0.9^A^	19.6 ± 0.5^A^
Ash – FW (g/100 g)	1.1 ± 0.2^A^	1.1 ± 0.1^A^	1.1 ± 0.1^A^
Ash – DM (g/100 g)	5.8 ± 0.6^A^	5.5 ± 0.5^A^	5.6 ± 0.5^A^
Fat – FW (g/100 g)	0.5 ± 0.0^A^	0.5 ± 0.1^A^	0.5 ± 0.1^A^
Fat – DM (g/100 g)	2.5 ± 0.3^A^	2.6 ± 0.5^A^	2.5 ± 0.3^A^
Protein – FW (g/100 g)	2.0 ± 0.2^A^	1.9 ± 0.3^A^	1.9 ± 0.3^A^
Protein – DM (g/100 g)	10 ± 1^A^	9 ± 1^A^	9 ± 2^A^
Total carbohydrate – FW (g/100 g)	16.0 ± 0.8^A^	16.0 ± 0.8^A^	16.1 ± 0.4^A^
Total carbohydrate – DM (g/100 g)	81.4 ± 0.9^A^	82 ± 2^A^	82 ± 2^A^

a T1 = dairy beverage control, without lactobacilli adjunct; T2 = dairy beverage with *L. rhamnosus* LR32; T3 = dairy beverage with *L. casei* BGP93. FW = fresh weight sample. DM = dry matter basis. ^A^In a row, trials sharing the same superscript uppercase letter did not significantly differ for the same parameter (*p* > 0.05).

### 3.2. Values of pH and viability of the starter and adjuvant cultures in the dairy bases during fermentation

**Fig. 1:**
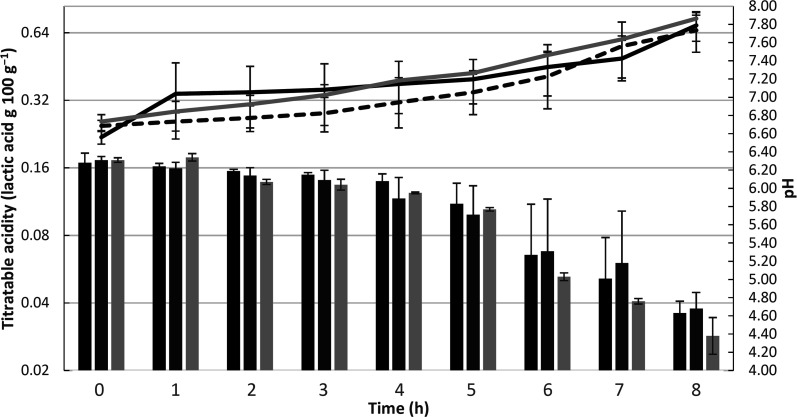
Changes in mean values of pH (bars) and titratable acidity (lines) during the fermentation process of the dairy bases from control T1 (black), added of *L. rhamnosus* T2 (dashed line and dark gray bar) and added of *L. casei* T3 (light gray). The error bars represent the standard deviation.

Changes in the mean values of pH and acidity of the dairy bases during the fermentation process are shown in Fig. 1. Significant differences between the treatments and fermentation times are presented in ESI 1.† During the fermentation process, there were significant differences (*p* > 0.05) in the pH value among trials in a same sampling period. The control dairy base (T1) and the dairy base with *L. casei* (T3) differed from the second to fourth hour of fermentation, while the *L. rhamnosus* base (T2) differed from the base T3 only at the seventh hour of fermentation.

In relation to the fermentation time, there was a significant reduction (*p* < 0.05) in pH throughout the process. For base T1, the pH was stable only between the beginning and the first hour, decreasing progressively over subsequent hours. Base T2 showed a decrease in pH from the beginning to the end of fermentation, and in base T3, pH decay also occurred continuously, except between the second and third hour, during which it was stable. At the end of the fermentation process, the T1, T2, and T3 dairy bases achieved a mean pH value of 4.6 ± 0.1, 4.7 ± 0.2, and 4.4 ± 0.2, respectively.

**Fig. 2:**
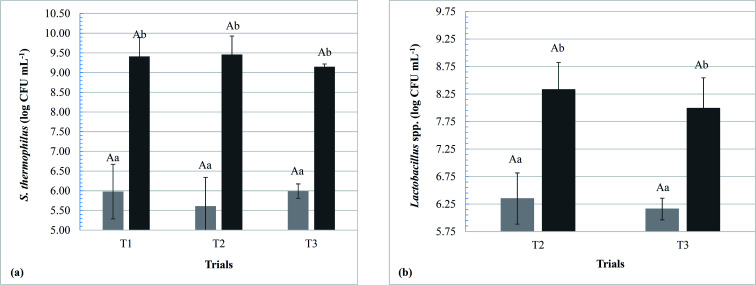
Populations of *S. thermophilus* in T1 (control), T2 (with *L. rhamnosus* LR32) and T3 (with *L. casei* BGP93) (a) and *Lactobacillus* spp. in the trials T2 and T3 (b) at times zero (light gray) and final time (dark gray) of the fermentation of the dairy bases. Different upper case letters denote significant differences between the assays for the same fermentation time and microorganism (*p* < 0.05). Different lowercase letters denote significant differences between fermentation times for the same assay and microorganism (*p* < 0.05).

The dairy bases containing *Lactobacillus* spp. started the fermentation (T0) with a significant difference (*p* < 0.05) from the control dairy base (T1); however, at the end of fermentation (TF), no significant differences were observed in this parameter (*p* > 0.05). Within the same trial, there was a significant increase in acidity with increasing fermentation time, ending with an acidity (g 100 g^−1^) of 0.7 ± 0.1, 0.7 ± 0.1, and 0.7 ± 0.0 for T1, T2, and T3, respectively. Populations of *S. thermophilus* and *Lactobacillus* spp. at the beginning and end of fermentation are shown in Fig. 2. The populations of the starter culture (*S. thermophilus*) increased significantly at the end of fermentation (*p* < 0.05) within the same trial; however, there were no significant differences among the different dairy bases (T1, T2, and T3), indicating that the addition of the adjuvant lactobacilli did not influence the viability of *S. thermophilus*.

The populations of the adjuvant lactobacilli added to T2 and T3 dairy bases did not differ significantly during fermentation; however, both presented a significant increase between time zero and the end of the process (*p* < 0.05), with a mean (in log CFU g^−1^) of 8.3 ± 0.5 for T2 and 8.0 ± 0.6 for T3. Similar results were found by the present research group for *L. rhamnosus* LR32 submitted to the fermentative process in milk-whey bases of cow^10^ and goat^11^ origin and also for both *L. rhamnosus* LR32 and *L. casei* BGP93 fermented in reconstituted goat whey powder,^16^ all of them with *S. thermophilus* TA-40 as starter culture. Moreover, Santos and co-workers^16^ verified that both co-cultures of *L. rhamnosus* LR32 or *L. casei* BGP93 with *S. thermophilus* were able to exert proteolytic activity on goat whey proteins during the fermentation process. According to the authors, proteinases and peptidases from starter and probiotic cultures are important to these microorganisms to obtain essential amino acids for their growth. More details on the proteolytic activity of these cultures in the products of the present study are discussed later.

### 3.3. Physicochemical parameters and viability of the starter and adjuvant cultures during the storage of the dairy beverage containing jambolan pulp

Changes in the mean pH values, titratable acidity, and populations of *S. thermophilus* and *Lactobacillus* spp. of the dairy beverages during the storage period are shown in Table 2. Beverages T1, T2, and T3 did not differ significantly during the storage period (*p* > 0.05), with the exception of T1 and T3, which differed significantly only on day 14. Similarly, the titratable acidity only differed significantly between T1 and T3 trials on day 14 and between beverages containing *Lactobacillus* spp. (T2 and T3) on day 21. On the other hand, post-acidification was observed in the products, since the pH decreased significantly in all trials (*p* < 0.05) during storage. This phenomenon occurs due to the active metabolism of the added cultures,^30^ and has been associated with textural changes^31^ and a reduction in the viability of probiotic bacteria.^32^ Regarding the storage period for each trial, there were significant differences between days 14 and 21 in beverages T1 and T3. However, although the acidity increased during the storage of trials, it did not affect the viability of the adjuvant lactobacilli, which remained higher than 7 log CFU g^−1^ in beverages T2 and T3.

**Table 2:** Changes in mean values of pH, titratable acidity and in populations of *S. thermophilus* and *Lactobacillus* spp. of the fermented dairy beverage trials after 1, 7, 14 and 21 days of storage at 4 ± 1 °C^a^

Parameters	Time (days)	Trials		
		T1	T2	T3
pH	D1	4.9 ± 0.3^Ad^	4.9 ± 0.4^Ac^	4.6 ± 0.3^Ac^
	D7	4.7 ± 0.3^Ac^	4.7 ± 0.5^Ac^	4.4 ± 0.1^Ab^
	D14	4.5 ± 0.3^Bb^	4.5 ± 0.3^ABb^	4.3 ± 0.1^Aa^
	D21	4.4 ± 0.3^Aa^	4.4 ± 0.2^Aa^	4.3 ± 0.1^Aa^
Titratable acidity (lactic acid g/100 g)	D1	0.6 ± 0.0^Aac^	0.6 ± 0.0^Aa^	0.7 ± 0.1^Ab^
	D7	0.7 ± 0.0^Aa^	0.7 ± 0.0^Aa^	0.6 ± 0.0^Ab^
	D14	0.7 ± 0.1^Aab^	0.7 ± 0.1^ABa^	0.6 ± 0.0^Bb^
	D21	0.6 ± 0.1^ABa^	0.6 ± 0.0^Ba^	0.6 ± 0.0^Aa^
*S. thermophilus* (log CFU g^−1^)	D1	9.3 ± 0.7^Aa^	8.8 ± 0.4^Aa^	9.3 ± 0.4^Abc^
	D7	9.0 ± 0.1^Aa^	9.0 ± 0.5^Aab^	8.9 ± 0.3^Aa^
	D14	9.0 ± 0.4^Aa^	9.0 ± 0.3^Ab^	9.2 ± 0.1^Ac^
	D21	9.1 ± 0.4^Aa^	9.0 ± 0.4^Ab^	8.9 ± 0.4^Aab^
*Lactobacillus* spp. (log CFU g^−1^)	D1	n.a	7.5 ± 0.5^Aa^	8 ± 1^Aa^
	D7	n.a	8.3 ± 0.3^Ab^	8 ± 1^Aab^
	D14	n.a	8.4 ± 0.3^Ac^	7.5 ± 0.8^Abc^
	D21	n.a	8.4 ± 0.3^Ac^	8 ± 1^Ac^

a T1 = dairy beverage control, without lactobacilli adjunct; T2 = dairy beverage with *L. rhamnosus* LR32; T3 = dairy beverage with *L. casei* BGP93. n.a. = not added. ^A,B^In a row, different superscript uppercase letters denote significant differences between trials for the same storage day (*p* < 0.05). ^a,b,c,d^In a column, different superscript lowercase letters denote significant differences between the storage days for the same trial in a same parameter (*p* < 0.05).

In the present study, there was no significant difference between the T2 and T3 trials regarding the populations of *Lactobacillus* spp. (*p* > 0.05). However, in relation to the storage time, there was a significant increase in their viability from day 1 to 14 (*p* < 0.05) for both trials, remaining stable at day 21 only for beverage T2 (*p* > 0.05). A significant decrease in the population of *Lactobacillus* spp. in beverage T3 was observed on day 21 as compared with day 1 (*p* < 0.05). It has been reported that the addition of fruit juices and pulps may influence the stability of probiotic microorganisms, for *e.g.*, Vinderola and co-workers^33^ evaluated the influence of fruit juices on probiotic cultures, and the results showed that the acidity of strawberry caused an inhibition of *Lactobacillus acidophilus* CNRZ 1881 and *Bifidobacterium longum* A1, but not strains in the *L. casei* group (four strains of *Lactobacillus paracasei*, three strains of *L. rhamnosus*, and one strain of *L. casei*). In the present study, however, *L. casei* BGP93 was more sensitive than *L. rhamnousus* LR32 to changes in acidity over the storage period. Céspedes and co-workers^34^ reported that the strain *L. casei* BGP93 show a cell decay of one log cycle by the 4th week in a commercial non-dairy matrix (multifruits Ades juice), which had achieved viability of approximately 8 log CFU mL^−1^ during the previous weeks. On the other hand, Buriti and co-workers^11^ verified that *L. rhamnosus* LR32 achieved populations of about one log cycle higher than that found for the probiotic *Bifidobacterium animalis* subsp. *lactis* BB-12 at 14 and 21 days of storage in goat dairy beverages in the presence of fruits (guava or graviola pulps), since the viability of both microorganisms had been close to 8 log CFU g^−1^ on the first day. In the present study, however, populations of *L. casei* BGP93 achieved values close to that reported by Pereira and co-workers^13^ for the same strain in sweetened goat dairy beverages without addition of fruits, also higher than 7 log CFU g^−1^ for 21 days of storage.

Based on human clinical trials reported by Martinez and co-workers^35^ in their review, the concentration of probiotic bacteria in a food able to perform health-promoting activities ranged from 10^6^ to 10^8^ CFU g ^−1^ food or 10^8^ to 10^10^ CFU per day. Values around 7.5 and 8 CFU g^−1^ lactobacilli were achieved throughout the storage period for beverages T2 and T3 in the present study, which may allow their characterisation as potentially probiotic products.

The populations of *S. thermophilus* did not differ among the three trials within each sampling period (*p* > 0.05), indicating that the adjuvant cultures did not interfere with the viability of the starter microorganism during storage.

### 3.4. Sensory evaluation of the fermented dairy beverage

A total of 128 judges participated in the present study, of which 49 were men and 79 were women, aged between 19 and 56 years old. Overall acceptability did not differ significantly among beverages evaluated on any day of storage, and no significant differences (*p* > 0.05) were observed in the same assay during storage (Table 3).

**Table 3:** Overall acceptability values (average ± standard deviation) obtained in the sensory analysis with judges (*n* = 128) for the fermented dairy beverages trials after 7, 14 and 21 days storage^a^

Attributes	Day	Trials		
		T1	T2	T3
Flavor	7	6 ± 3^Aa^	6 ± 3^Aa^	6 ±3^Aa^
	14	6 ± 2^Aa^	6 ± 3^Aa^	6 ±2^Aa^
	21	7 ± 2^Aa^	6 ± 3^Aa^	7 ±2^Aa^
Sweetness	7	6 ± 2^Aa^	6 ± 2^Aa^	7 ± 2^Aa^
	14	6 ± 2^Aa^	6 ± 3^Aa^	7 ± 2^Aa^
	21	6 ± 2^Aa^	7 ± 3^Aa^	7 ± 2^Aa^
Consistency	7	6 ± 2^Aa^	7 ± 2^Aa^	7 ± 2^Aa^
	14	6 ± 2^Aa^	7 ± 2^Aa^	7 ± 2^Aa^
	21	7 ± 2^Aa^	7 ± 2^Aa^	7 ± 2^Aa^
Appearance	7	7 ± 2^Aa^	7 ± 2^Aa^	8 ± 2^Aa^
	14	7 ± 2^Aa^	8 ± 2^Aa^	8 ± 2^Aa^
	21	7 ± 2^Aa^	8 ± 2^Aa^	7 ± 2^Aa^
Color	7	8 ± 2^Ab^	8 ± 2^Aa^	8 ± 2^Aa^
	14	7 ± 2^Aa^	8 ± 2^Aa^	8 ± 2^Aa^
	21	8 ± 2^Aab^	8 ± 2^Aa^	7 ± 2^Aa^
General acceptability	7	7 ± 2^Aa^	7 ± 2^Aa^	7 ± 2^Aa^
	14	6 ± 2^Aa^	7 ± 2^Aa^	7 ± 2^Aa^
	21	7 ± 2^Aa^	7 ± 2^Aa^	7 ± 2^Aa^

a T1 = dairy beverage control, without lactobacilli adjunct; T2 = dairy beverage with *L. rhamnosus* LR32; T3 = dairy beverage with *L. casei* BGP93. ^A^In a row, same superscript uppercase letters did not differ significantly between the trials studied in a same day (*p* > 0.05). ^a^In a column, same lowercase letters did not differ significantly over time for the same trial in a same parameter (*p* > 0.05).

In the present study, it can be observed that the different cultures used in the dairy beverage did not result in significant changes in the evaluated sensorial attributes. In studies by Almeida Neta and co-workers^10^ and Pereira and co-workers,^36^ strains of *L. rhamnosus* LR32 and *L. casei* BGP93, respectively, were used as adjuvant cultures with *S. thermophilus* TA-40, and the results of sensory analysis indicated that such *Lactobacillus* spp. did not change the acceptability of the tested products (jabuticaba fermented dessert and goat dairy beverage, respectively, both formulated with whey).

Sampling periods did not interfere with the overall acceptability by the judges, since the average scores awarded were close across all times and trials, and therefore, did not result in significant changes that could be perceived through sensory evaluation (*p* > 0.05). When assessing the opinion of the judges in relation to the sensorial attributes that were more and less appreciated, it was observed that the flavour and consistency presented more citations as “less appreciated”, whereas the colour was the attribute that obtained more citations as “more appreciated” (data not shown).

The lower citations for flavour as “more appreciated” may be related to the lack of familiarity of the judges with this fruit, since it has no commercial use and jambolan is not yet used in dairy products and the purple colour is associated with grape products, which likely reinforced the strangeness of the exotic flavour of a product with this colour. Regarding the lower citations for consistency as “more appreciated”, it is important to highlight that, in general, milk-whey-based beverages have a more fluid consistency than yogurts.^11^ It is likely that the judges assumed that the dairy beverages in the present study should have similar characteristics to a yogurt, and thus expected a firmer and creamier product.

On the other hand, colour was the attribute cited as “most appreciated” in all beverages and at all storage times, rather a positive result since no artificial colourants were used in the product and its colour was a result only of the addition of the jambolan pulp, which according to Branco and co-workers,^6^ is a fruit rich in anthocyanins, conferring the purplish colour. Torskangerpoll and co-workers^37^ reported that anthocyanins may undergo changes in pH, and consequently change colour; however, in the present study, the colour of the products was shown to be stable even with variation in the acidity during storage.

### 3.5. Total phenolic content and antioxidant capacity of dairy beverages containing jambolan pulp

The total phenolic content (in mg GAE 100 g^−1^), as well as the percentage inhibition of DPPH radicals, the EC_50_, and the antioxidant capacity of the sample (in g DPPH per g sample) for the dairy bases and beverages T1, T2, and T3 over the storage period at 4 ± 1 °C are shown in Table 4.

**Table 4:** Total phenolic content, EC_50_, percent of DPPH radicals scavenging and total antioxidant capacity of the dairy bases before (T0) and after (TF) fermentation and their respective fermented dairy beverages trials after 1, 7, 14 and 21 days of storage at 4 ± 1 °C^a^

Parameters	Sampling periods	Trials		
		T1	T2	T3
Total phenolics (mg GAE per 100 g)	T0	16 ± 5^Aa^	22 ± 8^Aa^	18 ± 3^Aa^
	TF	17 ± 3^Aa^	19 ± 10^Aa^	19 ± 3^Ab^
	D1	42 ± 10^Ab^	36 ± 6^Ab^	44 ± 3^Ac^
	D7	40 ± 8^Ab^	37 ± 8^Ab^	37 ± 3^Ad^
	D14	38 ± 4^Ab^	43 ± 11^Ab^	36 ± 5^Ad^
	D21	39 ± 12^Ab^	37 ± 3^Ab^	36 ± 4^Ad^
EC_50_ (g sample per L DPPH 100 μM)	T0	41 ± 6^Ad^	61 ± 19^ABe^	50 ± 2^Bd^
	TF	37 ± 3^Ac^	81 ± 10^Bf^	41 ± 17^Ad^
	D1	18 ± 1^Ab^	17.0 ± 0.9^Ac^	18 ± 1^Ac^
	D7	13.9 ± 0.3^Ba^	21 ± 3^Cd^	12.0 ± 0.5^Ab^
	D14	14 ± 2^Ba^	14.0 ± 0.5^Ba^	10.4 ± 0.2^Aa^
	D21	16 ± 4^ABab^	15.0 ± 0.4^Bb^	12.1 ± 0.3^Ab^
Total antioxidant capacity (g sample per g DPPH)	T0	1178 ± 300^Ac^	1815 ± 451^Aa^	1533 ± 683^Ae^
	TF	1433 ± 43^Ac^	2570 ± 279^Ba^	1972 ± 760^ABe^
	D1	791 ± 132^Ab^	954 ± 298^Ab^	789 ± 50^Ad^
	D7	633 ± 85^Aab^	970 ± 285^Bb^	624 ± 26^Ac^
	D14	615 ± 70^Aa^	539 ± 74^Aa^	525 ± 21^Ab^
	D21	696 ± 165^Aa^	577 ± 72^Aa^	609 ± 25^Aa^
DPPH radicals scavenging (%)	T0	8 ± 8^Aa^	9 ± 9^Aa^	15 ± 9^Aa^
	TF	5 ± 2^Aa^	3 ± 2^Aa^	7 ± 2^Aa^
	D1	21 ± 3^Ab^	24 ± 4^Ab^	27 ± 4^Ab^
	D7	25 ± 1^Ab^	24 ± 8^Ab^	37.6 ± 0.5^Ab^
	D14	26 ± 4^Ab^	28 ± 3^Ab^	40.2 ± 0.3^Ab^
	D21	25 ± 9^Ab^	25.2 ± 0.6^Ab^	30.9 ± 0.3^Ab^

a DPPH = 1,1-diphenyl-2-picrylhydrazyl; EC_50_ = amount of sample required to reduce the initial concentration of DPPH by 50%. T1 = control trial, without lactobacilli adjunct; T2 = trial with *L. rhamnosus* LR32; T3 = trial with *L. casei* BGP93. ^A,B^In a row, different superscript uppercase letters denote significant differences between trials for the same storage day (*p* < 0.05). ^a,b,c,d^In a column, different superscript lowercase letters denote significant differences between the storage days for the same trial in a same parameter (*p* < 0.05).

Analysis of the results obtained for the total phenolic content showed that the dairy bases (T0 and TF) presented values significantly lower than those of the beverage containing jambolan throughout storage (*p* < 0.05), which implies that the addition of the pulp of this fruit increased its phenolic content. Bezerra and co-workers^7^ evaluated the total phenolic content of four types of frozen yogurt that were differentiated by their composition of microorganisms (*Lactobacillus delbrueckii* subsp. *bulgaricus* and *Streptococcus thermophilus* Y540B with and without *Bifidobacterium animalis* subsp. *lactis* BI-07) and the fruit forms (powder or pulp), with averages between 5.25 and 9.03 mg g^−1^. The dairy beverage produced in the present study therefore presented total phenolic values higher than those shown by Bezerra and co-workers,^7^ however, it should be noted that the type of extraction employed for these compounds was different from that used in the present study.

According to Corrêa and co-workers,^38^ which estimated the consumption of phenolic compounds by the Brazilian population from the Brazilian Dietary Survey (Inquérito Nacional de Alimentação, INA) within the Household Budget Survey (Pesquisa de Orçamentos Familiares, POF), the Brazilian population consumes an average of 460.15 mg phenolic compounds per day. At the end of the storage period, the beverages produced in the present study had average values between 36 and 39 mg GAE per 100 g product, which represents approximately 8% of the daily phenolic consumption by the Brazilian population. Considering the 200 g serving established for dairy beverages by the current Brazilian regulatory standards,^29^ the beverage produced in the present study would offer 16% of the daily phenolic consumption.

In the present study, during the storage period, there were no significant differences (*p* > 0.05) in phenolic content among the beverages, except for the T3 trial between 1 and 7 days. However, evaluation of the results of EC_50_ showed that there was a significant reduction in the amount of beverage required to reduce the absorbance of the DPPH solution by half (*p* < 0.05) with an increase in the storage period (after 14 days for T2 and after 7 days for T1 and T3), indicating an increase in the antioxidant effect of the products throughout storage.

**Fig. 3:**
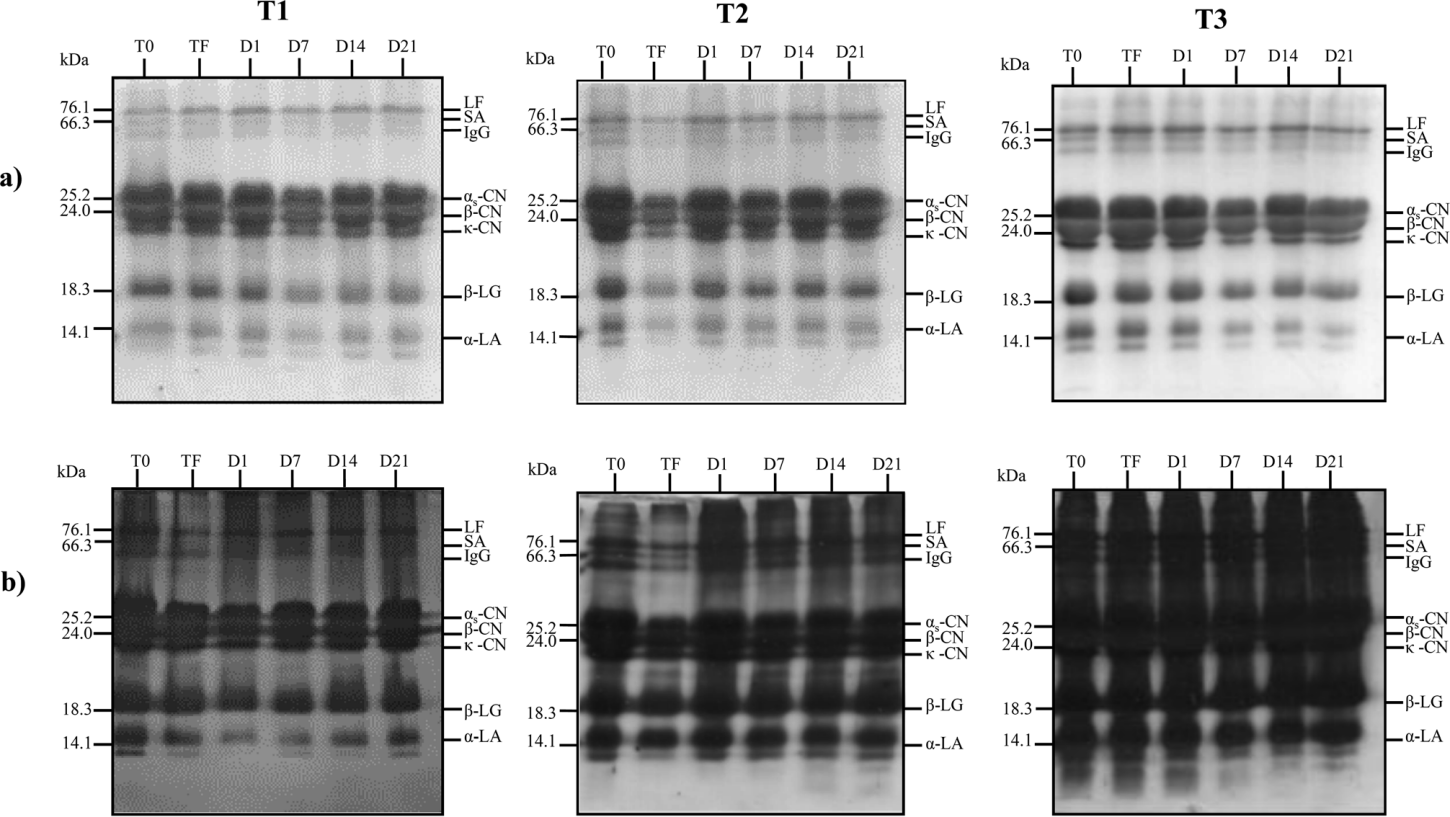
Electrophoretic characterization (SDS-PAGE) of proteins from dairy beverage stored at 4 ± 1 °C for 21 days. Gels (a) stained with Coomassie Brilliant Blue and gels (b) stained with silver nitrate. Each gel contains samples of the dairy base before (T0) and after (TF) fermentation and the corresponding dairy beverages, with 1, 7, 14, 21 storage days (D1, D7, D14, D21 respectively). T1 = control trial, without lactobacilli adjunct; T2 = trial with *L. rhamnosus* LR32; T3 = trial with *L. casei* BGP93.

Since there was no significant increase in the phenolic content of the products during storage, a significant reduction in EC_50_ within the storage period would likely be related to the additive effect of the jambolan and the occurrence of proteolysis, as observed in the SDS-PAGE gels (Fig. 3), with the possible release of bioactive fractions with antioxidant capacity.

Liu and co-workers^39^ investigated the ability of *L. rhamnosus* 6134 to affect the antioxidant activity of cheddar cheese. The results showed that addition of that strain increased the DPPH radical scavenging capacity and reducing power by 7.46% and 17.58%, respectively, as compared with cheeses without probiotic addition. These findings reinforce the idea that the addition of probiotics can improve peptide formation and antioxidant activity.

Through the evaluation of the total antioxidant capacity of the dairy beverages produced in the present study, it is possible to emphasise the strong influence of the addition of jambolan pulp to the products, since between 1178 and 1815 g dairy bases in T0 (without addition of the fruit) was needed to scavenge 1 g DPPH radicals, whereas for the dairy beverage containing pulp (D1), the average amount of sample needed was 790, 953, and 789 g for T1, T2, and T3, respectively. The possible activity of protein fractions with antioxidant properties is highlighted again, considering that the amount of dairy beverage needed to scavenge 1 g DPPH continued to decrease over the storage period.

In relation to the percentage inhibition of DPPH radicals (%), the mean values obtained in the present study for all dairy beverages containing jambolan pulp in the maximum aliquot of sample extract used in the test (0.2 mL sample extract for a volume of 3 mL with 100 μM DPPH) were superior to 20% inhibition of DPPH radicals and were significantly higher than the mean values found for the fermented bases before the addition of jambolan (TF), reinforcing the influence of pulp fruit on the antioxidant capacity of the produced dairy beverages. Almeida Neta and co-workers^10^ obtained averages above 30% inhibition of DPPH radicals in milk-whey based desserts fermented with *S. thermophilus* TA-40 co-cultured with *L. rhamnosus* LR32 or *Lactobacillus plantarum* CNPC003 and added of jabuticaba peel. However, the authors incorporated jabuticaba peel syrup and jam into the formulations, as well as hydroethanolic extract obtained from the peel, while in the present study, only the pulp of jambolan was used. Studies with pineapple waste powder^40^ and grape pomace^5^ have also highlighted that the use of fruits improves the antioxidant activity of dairy products.

### 3.6. Analysis of the protein profile of the fermented dairy beverages by SDS-PAGE

The main proteins of cow milk, LF, SA, immunoglobulin G, α_s_-CN, β-CN, κ-CN, β-LG and α-LA,^26^ were revealed in the gels for all samples analyzed, for both staining methods used (Fig. 3). Although the EC_50_ values reduced significantly during the storage for T1 trial (Table 4), the proteolytic activity within this period was not so evident in the T1 trial. In the T2 trial, with the presence of *L. rhamnosus*, it was possible to verify the formation of peptides in the region below 14 kDa on days 14 and 21 of the storage only in the gel stained with silver nitrate, since this staining method has high sensitivity, being able to detect protein fragments of 0.1 to 1.0 ng, whereas Coomassie Brilliant staining detects proteins of 30 to 100 ng.^41^ On the other hand, in the T3 trial, with *L. casei*, proteolytic activity on α-LA was observed in Coomassie Brilliant-stained gel. Moreover, the peptides present in T0, TF and D1 samples of T3 trial have been degraded throughout the storage time and their lower derived fractions could not be detected by silver nitrate staining. These results reinforce the possibility that such peptides formed in T2 and T3 beverages were related to the increase in the antioxidant capacity observed throughout storage, as mentioned previously. Sadat and co-workers^42^ verified that five peptides isolated from the hydrolysis of α-LA were able to show antioxidant activity by the ABTS method. Whey proteins were probably involved in the antioxidant results verified in the present study for T3 trial with *L. casei*. The strain *L. casei* BGP93, used in co-culture with *S. thermophilus* TA-40, have shown to result in proteolytic activity in goat whey-based beverages with inhibitory activity against angiotensin converting enzyme (ACE) in a previous study of our research group.^13^ In another study, Santos and co-workers^16^ verified that relative amount of low-molecular-weight protein fractions (<6.5 kDa) increased in reconstituted goat whey powder fermented with co-cultures of *S. thermophilus* TA-40 with *L. casei* BGP93 or *L. rhamnosus* LR32 stored for 7 days at 4 °C; however, according to the authors, the pattern of degradation of goat whey proteins during storage differed between those trials, particularly for fractions of 50–90 kDa that only reduced in the TA-40 plus BGP3 trial. Solieri and co-workers^43^ evaluated the proteolytic capacity of strains of *L. casei* PRA205 and *L. rhamnosus* PRA331 in fermented milk and observed the production of peptides, highlighting the specificity of each bacterial strain in the proteolysis profile, as shown in the present study.

In general, proteolysis in dairy products consists of protein degradation performed by endogenous milk enzymes and enzymes originating from lactic acid bacteria, which results in the production of low- and medium-molecular weight peptides and free amino acids. Peptides resulting from proteolytic activity on milk proteins are inactive when remaining within the original protein, but when released, they may exert beneficial health activities such as angiotensin converting enzyme inhibitory activity, and also opioid, antioxidant, antidiabetic, immunomodulatory, and antimicrobial activities.^44^

Dairy products are generally considered rich sources of bioactive peptides, which can positively modulate physiological and metabolic functions.^45^ The incorporation of lactic acid bacterial strains may promote the release of bioactive peptides due to their proteinase action. Ni and co-workers^46^ investigated the potential of the addition of extracts of salted berries (*Gaultheria shallon*) and blackcurrant (*Ribes nigrum*) to a yogurt matrix. A total of 486 peptides were isolated, of which 15 showed bioactivity predominantly as antimicrobial agents or angiotensin converting enzyme (ACE) inhibitors. Li and co-workers^47^ studied the ACE inhibitory activity of 41 strains of *L. casei* in the production of fermented milk, with 22 strains showing production of peptides inhibiting this enzyme. These studies reinforce the role of lactic acid bacteria in the production of bioactive peptides from the proteolysis of milk proteins.

## 4. Conclusions

The probiotic adjuvants slightly influenced the pH and titratable acidity of dairy beverages, with no influence on the proximate composition and on the sensory attributes. Moreover, the lactic cultures studied contributed for proteolysis in dairy beverages, which in an additive effect with the presence of jambolan pulp, were probably related to the significant decrease in EC_50_ of the products during the storage period. The described products represent an alternative use of cheese whey, considered a waste by the cheese industry despite its nutritional value, and the pulp of jambolan, a fruit that is still poorly explored, with low economic value and high prevalence in the region of study. Therefore, the studied dairy beverages are thus seen as good functional food options due to their nutritional value, viability of potentially probiotic lactobacilli, total phenolic content, and antioxidant activity. In addition, these beverages can serve the lactose-intolerant public by representing a functional dairy product option.

## Conflicts of interest

There are no conflicts to declare.

## Supplementary Material

RA-010-C9RA08311A-s001
