# Correction: Influence of co-cultures of *Streptococcus thermophilus* and probiotic lactobacilli on quality and antioxidant capacity parameters of lactose-free fermented dairy beverages containing *Syzygium cumini* (L.) Skeels pulp

**DOI:** 10.1039/d0ra90046j

**Published:** 2020-05-06

**Authors:** Sabrina Laís Alves Garcia, Gabriel Monteiro da Silva, Juliana Maria Svendsen Medeiros, Anna Paula Rocha de Queiroga, Blenda Brito de Queiroz, Daniely Rayane Bezerra de Farias, Joyceana Oliveira Correia, Eliane Rolim Florentino, Flávia Carolina Alonso Buriti

**Affiliations:** Post-Graduate Program on Pharmaceutical Sciences, Centre of Biological and Health Sciences, State University of Paraíba R. Juvêncio Arruda, s/n, 58429-600 Campina Grande PB Brazil flavia@ccbs.uepb.edu.br flavia.carolina@pq.cnpq.br; Centre of Research and Extension on Food, Centre of Sciences and Technology, State University of Paraíba R. Juvêncio Arruda, s/n, 58109-790 Campina Grande PB Brazil; Department of Chemistry, Centre of Sciences and Technology, State University of Paraíba R. Juvêncio Arruda, s/n, 58109-790 Campina Grande PB Brazil; Department of Pharmacy, Centre of Biological and Health Sciences, State University of Paraíba R. Juvêncio Arruda, s/n, 58429-600 Campina Grande PB Brazil

## Abstract

Correction for ‘Influence of co-cultures of *Streptococcus thermophilus* and probiotic lactobacilli on quality and antioxidant capacity parameters of lactose-free fermented dairy beverages containing *Syzygium cumini* (L.) Skeels pulp’ by Sabrina Laís Alves Garcia *et al.*, *RSC Adv.*, 2020, **10**, 10297–10308. DOI: 10.1039/c9ra08311a

The authors would like to correct an error in the “Materials and methods” section which they noticed after publication of their article.

In the first sentence of the first paragraph of Section 2.7. Sensory evaluation of the fermented dairy beverage, “Certificate of Presentation for Ethical Assessment (CAAE) no. 2.229.0000.5187” should read “Certificate of Presentation for Ethical Assessment (CAAE) no. 71428417.5.0000.5187, decision no. 2.229.941”.

The first sentence of the first paragraph of Section 2.7. Sensory evaluation of the fermented dairy beverage incorporating the correction is as follows:

“The sensory evaluation used in the present study was approved by the Ethics Committee of the State University of Paraíba (UEPB), Paraíba, Brazil, Certificate of Presentation for Ethical Assessment (CAAE) no. 71428417.5.0000.5187, decision no. 2.229.941, and was performed in the Laboratory of Sensory Analysis at the Federal University of Campina Grande (UFCG), Paraíba State, Brazil.”

The Royal Society of Chemistry apologises for these errors and any consequent inconvenience to authors and readers.

## Supplementary Material

