# Supplementary material for: Influence of co-cultures of Streptococcus thermophilus and probiotic lactobacilli on quality and antioxidant capacity parameters of lactose-free fermented dairy beverages containing Syzygium cumini (L.) Skeels pulp
Source: RSC Adv. 2020 Mar 10;10(17):10297–308. doi: 10.1039/c9ra08311a (PMC9050390; doi:10.1039/c9ra08311a)
Supplement: RA-010-C9RA08311A-s001 [file RA-010-C9RA08311A-s001.pdf]

Electronic Supplementary Material (ESI) for RSC Advances.
This journal is © The Royal Society of Chemistry 2020

Supplementary material

Table S1. Statistical analysis (mean±standard deviation) for the pH and titratable acidity of formulations during the fermentation process.

Time (h)	T1		T2		T3	
	Lactic acid ($g\ 100\ g^{-1}$)	pH	Lactic acid ($g\ 100\ g^{-1}$)	pH	Lactic acid ($g\ 100\ g^{-1}$)	pH
0	0.2 ± 0.0^{Aa}	6.3 ± 0.1^{Agh}	0.2 ± 0.0^{Ba}	6.3 ± 0.0^{Ai}	0.3 ± 0.0^{Ba}	6.3 ± 0.0^{Ah}
1	0.3 ± 0.1^{Abc}	6.2 ± 0.0^{Ah}	0.3 ± 0.0^{Ab}	6.2 ± 0.1^{Ah}	0.3 ± 0.0^{Ab}	6.3 ± 0.0^{Bg}
2	0.3 ± 0.1^{Ac}	6.2 ± 0.0^{Bg}	0.3 ± 0.0^{Ab}	6.1 ± 0.1^{ABg}	0.3 ± 0.0^{Ac}	6.1 ± 0.0^{Af}
3	0.4 ± 0.1^{Acd}	6.2 ± 0.0^{Bf}	0.3 ± 0.0^{Ac}	6.1 ± 0.1^{ABf}	0.3 ± 0.0^{Ad}	6.0 ± 0.1^{Af}
4	0.4 ± 0.1^{Ab}	6.1 ± 0.1^{Be}	0.3 ± 0.1^{Ad}	5.9 ± 0.2^{ABe}	0.4 ± 0.0^{Ae}	6.0 ± 0.0^{Ae}
5	0.4 ± 0.1^{Ab}	5.8 ± 0.2^{Ad}	0.3 ± 0.1^{Ae}	5.7 ± 0.3^{Ad}	0.4 ± 0.0^{Af}	5.8 ± 0.0^{Ad}
6	0.5 ± 0.1^{Abd}	5.3 ± 0.6^{Ac}	0.4 ± 0.1^{Ae}	5.3 ± 0.6^{Ac}	0.5 ± 0.0^{Ag}	5.0 ± 0.0^{Ac}

T1 = dairy beverage control, without lactobacilli adjunct; T2 = dairy beverage with *L. rhamnosus* LR32; T3 = dairy beverage with *L. casei* BGP93. $^{A, B}$ = In a row, different superscript uppercase letters denote significant differences between trials for the same fermentation time ($p < 0.05$). $^{a, b, c, d, e, f, g}$ = In a column, different superscript lowercase letters denote significant differences between the fermentation time for the same trial in a same parameter ($p < 0.05$).
